# P-1154. Epidemiology of pediatric invasive infections in a tertiary university hospital, 2014-2023, Daejeon, Republic of Korea

**DOI:** 10.1093/ofid/ofae631.1340

**Published:** 2025-01-29

**Authors:** Hyejin So, Kyung Min Kim, Eun Young Cho

**Affiliations:** Chungnam National University Sejong Hospital, Sejong, Ch'ungch'ong-bukto, Republic of Korea; Chungnam National University Hospital, Daejeon, Taejon-jikhalsi, Republic of Korea; Chungnam National University Hospital, Daejeon, Taejon-jikhalsi, Republic of Korea

## Abstract

**Background:**

In early 2010s, vaccines against pneumococcus and *H. influenzae* have been introduced in Korea as a national vaccination, reducing the burden of pediatric invasive disease in the community. However, invasive infections in children and adolescents with underlying diseases remains important and to provide effective antibiotic treatment is essential.

**Figure:**
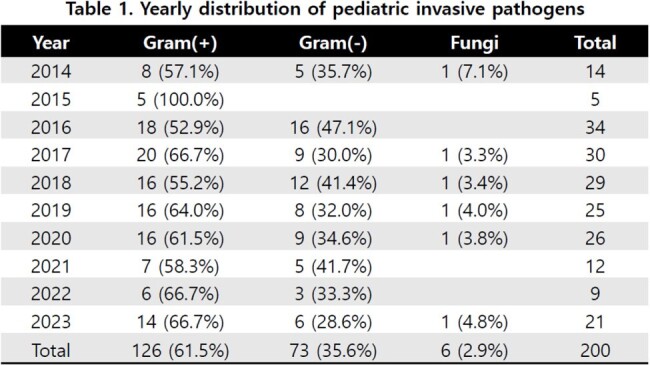

**Methods:**

To search the epidemiology of invasive infection in children and adolescents, we analyzed culture results from sterile body fluids (blood, cerebrospinal fluid, pleural fluid, joint fluid) of aged 1 month through 18 years who were hospitalized in a tertiary university hospital in Daejeon from 2014 to 2023.

**Figure:**
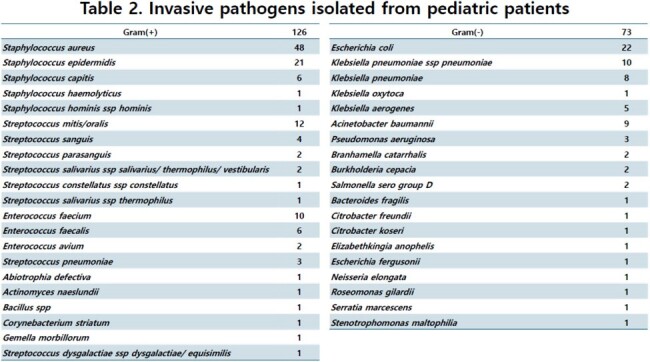

**Results:**

A total of 205 pathogens were cultured, most of which were isolated from patients with underlying diseases. Among the strains, 62% were Gram-positive bacteria, 36% were Gram-negative bacteria, and 3% were fungi. Among the 126 Gram-positive bacteria, 48 (38%) were *S. aureus*, 29 (23%) were coagulase negative staphylococci, 18 (14%) were enterococcus, and 3 isolates were *S. pneumoniae*. Among 73 Gram-negative bacteria, 22 were *E. coli* (30%), 19 were *K. pneumoniae* (26%), and 2 *Salmonella* were isolated. Among *S. aureus*, methicillin and clindamycin resistance were 52% and 48%, respectively, while vancomycin-resistance was not found. Among *E. coli* and *K. pneumoniae*, 42% of isolates were ESBL-producing strains. Resistance to cefotaxime, piperacillin/tazobactam, carbapenem were 49%, 20%, 5%, respectively.

**Figure:**
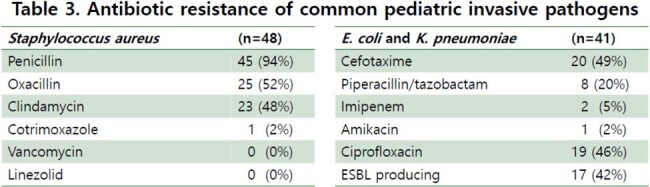

**Conclusion:**

Bacterial cultures in children and adolescents remains very important since it provides antibiotic susceptibility data, leading to appropriate treatment. Surveillance of invasive infections in children and adolescents should be continued in the era of increasing antibiotic resistance.

**Disclosures:**

**All Authors**: No reported disclosures

